# Assessing anthropogenic impact on boreal lakes with historical fish species distribution data and hydrogeochemical modeling

**DOI:** 10.1111/gcb.12527

**Published:** 2014-03-21

**Authors:** Salar Valinia, Göran Englund, Filip Moldan, Martyn N Futter, Stephan J Köhler, Kevin Bishop, Jens Fölster

**Affiliations:** 1 Department of Aquatic Sciences and Assessment, Swedish University of Agricultural SciencesBox 7050, Uppsala, SE-750 07, Sweden; 2 Department of Ecology and Environmental Science, Umeå UniversityUmeå, SE-901 87, Sweden; 3 Department of Earth Sciences, Uppsala UniversityVillavägen 16, Uppsala, 752 36, Sweden; 4 IVL Swedish Environmental Research InstituteBox 53021, Göteborg, SE-400 14, Sweden

**Keywords:** anthropogenic impact, boreal lakes, fish species composition, hydrogeochemical modeling, long-term observations, reference condition, undisturbed state

## Abstract

Quantifying the effects of human activity on the natural environment is dependent on credible estimates of reference conditions to define the state of the environment before the onset of adverse human impacts. In Europe, emission controls that aimed at restoring ecological status were based on hindcasts from process-based models or paleolimnological reconstructions. For instance, 1860 is used in Europe as the target for restoration from acidification concerning biological and chemical parameters. A more practical problem is that the historical states of ecosystems and their function cannot be observed directly. Therefore, we (i) compare estimates of acidification based on long-term observations of roach (*Rutilus rutilus*) populations with hindcast pH from the hydrogeochemical model MAGIC; (ii) discuss policy implications and possible scope for use of long-term archival data for assessing human impacts on the natural environment and (iii) present a novel conceptual model for interpreting the importance of physico-chemical and ecological deviations from reference conditions. Of the 85 lakes studied, 78 were coherently classified by both methods. In 1980, 28 lakes were classified as acidified with the MAGIC model, however, roach was present in 14 of these. In 2010, MAGIC predicted chemical recovery in 50% of the lakes, however roach only recolonized in five lakes after 1990, showing a lag between chemical and biological recovery. Our study is the first study of its kind to use long-term archival biological data in concert with hydrogeochemical modeling for regional assessments of anthropogenic acidification. Based on our results, we show how the conceptual model can be used to understand and prioritize management of physico-chemical and ecological effects of anthropogenic stressors on surface water quality.

## Introduction

Defining targets for restoration of impacted ecosystems in a changing world is challenging. Humans have impacted their supporting ecosystems throughout history, making any definition of undisturbed reference states ambiguous. Environmental legislation including the EU Water Framework Directive (WFD; 2000/60/EC) and US Clean Water Act are nevertheless based on the assumption that undisturbed reference conditions can be identified. The pragmatic solution to this dilemma has been to specify a point in time during which human impacts were considerably lower than today. For example, 1860 is widely used in Europe as the point in time when undisturbed reference conditions existed. While the impacts of fossil fuel burning on surface water acidification at this time were minor, 1860 is not truly indicative of undisturbed reference conditions as agricultural activities were causing increased pH values (Renberg *et al.*, 2009). A more practical problem is that the historical states of ecosystems and their function cannot be observed directly. Reconstructions of reference conditions are therefore typically based on sediment records, mechanistic models, or studies of presumed undisturbed sites; all of which are associated with inaccuracies. In this paper, we illustrate these problems using data on acidification of freshwaters.

Deposition of acidifying sulfur and nitrogen has caused severe effects on acid-sensitive freshwater ecosystems in Northern Europe and North America (Schindler, 1988). The peak of acid anion concentration in precipitation was reached during 1970–1980 (Mylona, 1993). Since then, acid deposition has declined in response to international legislative efforts, including the Convention on Long-Range Transboundary Air Pollution (UNECE, 2012) in Europe and the Clean Air Act in North America. Evidence of chemical recovery has been identified in regions of Europe and North America (Stoddard *et al.*, 1999; Evans *et al.*, 2001). Full chemical recovery is, however, expected to take decades (Wright *et al.*, 2005; Moldan *et al.*, 2013) and biological recovery appears to be patchy and without clear trends (Tipping *et al.*, 2002; Skjelkvale *et al.*, 2003; Angeler & Johnson, 2012).

An important effect of acidification is the loss of sensitive fish species, such as roach (*Rutilius rutilius*), Atlantic salmon (*Salmo salar*) and Brown trout (*Salmo trutta*) (Schofield, 1976; Rask *et al.*, 1995; Hesthagen *et al.*, 1999). Negative effects on roach were observed in the 1960s in Swedish lakes (Almer *et al.*, 1974) and effects on brown trout were observed as early as 1920s in southern Norway (Dahl, 1921). Reproductive failure and extinction of roach typically occurs in the pH range 5.0–6.0 and pH 5.5 is often considered as a threshold value for roach extinction (Vuorinen *et al.*, 1993; Holmgren & Buffam, 2005). Low pH *per se* very rarely causes fish death. Fish mortality is primarily controlled by pH-related changes in Aluminium (Al) solubility and speciation (Driscoll *et al.*, 1980; Baker & Schofield, 1982; Gensemer & Playle, 1999). At pH<5.5, there is a rapid increase in the concentrations of inorganic monomeric forms of Al (Al_i_), which are associated with fish mortality, (Driscoll & Schecher, 1990; Huser & Rydin, 2005).

Efforts to restore acidified freshwaters in Europe are guided by the WFD (EC, 2000). The overall goal of this directive is ‘good ecological status’, which is defined as an undisturbed reference state (with minor deviations accepted). In Sweden, reference states for surface water acidification are inferred using a hydrogeochemical model, MAGIC (Model of Acidification of Groundwater in Catchments) (Cosby *et al.*, 2001; SEPA, 2007; Moldan *et al.*, 2013). This model is used to reconstruct (or ‘hindcast’) lake water chemistry in 1860 based on contemporary lake water and precipitation chemistry, land use, deposition history, and hydrology (Cosby *et al.*, 2001). The 1860 reference state is compared to contemporary conditions to determine the deviation (ΔpH) from reference conditions. A contemporary pH<0.4 units below the 1860 reference pH is regarded as the threshold value for good ecological status in Sweden related to significant biological change (Fölster *et al*., 2007; SEPA, 2007).

The ΔpH approach is a major improvement of earlier methods based on threshold values defined by the tolerance of indicator organisms (Fölster *et al*., 2007). The primary reason is that many boreal surface waters are naturally acidic as a result of high levels of dissolved organic matter (Erlandsson *et al.*, 2011). The depauperate biological communities in these systems do not reflect anthropogenic activities, but represent an undisturbed state that should not be subject to restoration (Dangles *et al.*, 2004). Attempts to validate predictions from the MAGIC model have used pH reconstruction based on diatom remains in sediments (Wright *et al.*, 1986; Jenkins *et al.*, 1990; Hinderer *et al.*, 1998; Stuchlík *et al*., 2002; Battarbee *et al.*, 2005; Erlandsson *et al.*, 2008a). These studies show good general agreement between the two methods, but have also identified classes of lakes for which model parameterization needs to be improved (e.g., Erlandsson *et al.*, 2008a). A weakness of the ΔpH approach is that changes in chemical criteria may not reflect the changes of valuable species that are the ultimate targets for restoration efforts. A substantial reduction in pH from a high level may mean that no critical pH-threshold is crossed, and thus that impacts on biota are minimal (Holt *et al.*, 2003). Likewise, it has been shown that a pH-change may have smaller biotic effects in naturally acidic waters than in water subjected to anthropogenic acidification (Petrin *et al.*, 2008). This suggests that much can be gained by combining the predictions of chemical change with approaches based on the tolerance of indicator species, i.e. by recording the historical and present distribution of species with known tolerances.

In this paper, we therefore (i) compare estimates of acidification based on long-term observations of roach (*Rutilus rutilus*) populations with hindcast pH time series from the MAGIC model; (ii) discuss policy implications and possible scope for use of long-term archival data for assessing human impacts on the natural environment; and (iii) present a novel conceptual model for interpreting the importance of physico-chemical and ecological deviations from reference conditions.

## Material and methods

Here, we present assessments of surface water acidification based on chemical and biological criteria. Implemented chemical criterion is the ΔpH>0.4 units from reference conditions. The biological criterion was based on presence and absence of roach before and after the period of high atmospheric acid deposition.

### Historical fish analysis (Roach)

Roach (*Rutilus rutilis*) is a good indicator of surface water acidification as it is ubiquitous in Swedish lakes when pH is above 6 and fails to reproduce and eventually is extirpated when pH is below 5.5 (Vuorinen *et al.*, 1993; Holmgren & Buffam, 2005). The effects of anthropogenic acid deposition on roach communities was one of the earliest and most important examples of biological response to acidification in Swedish lakes (Almer *et al.*, 1974).

Roach observations were obtained from the database PIKE, which includes presence/absence data for 55 fish species in *c*. 18 000 Swedish lakes (http://www.emg.umu.se/english/research/research-projects/pike/, assembled by G. Englund). Data were obtained through interviews, questionnaires distributed by post and gillnet surveys performed during the period 1890–2012, as well as large-scale inventories of more than 7000 lakes performed during 1890–1940.

**Fig 1:**
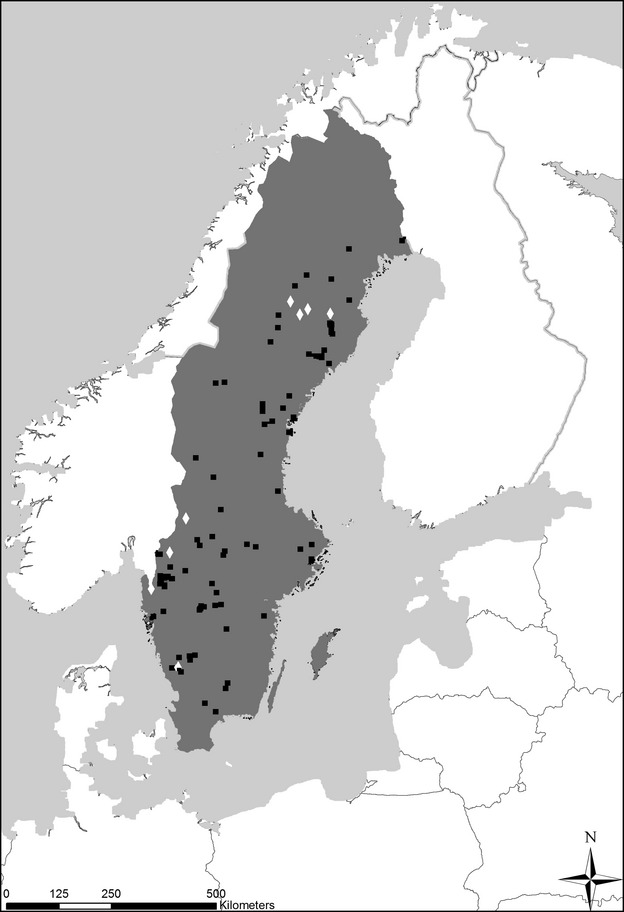
Spatial distribution of the 85 lakes was included in this study. Squares indicate were roach and MAGIC agree on the classification of good ecological status and diamonds indicate lakes where classifications based on MAGIC and the presence/absence of roach are inconsistent.

Data on presence/absence of roach were analyzed to determine if this species was present historically and if/when it went extinct during the acidification period. Observations were grouped into three periods: (i) ‘beginning of acidification period’ (before 1960), (ii) ‘heavy acidification period’ (1960–1990), and (iii)‘recovery period’ (1990–2012). Only lakes where roach was present during period 1 and with additional data from period 2 were analyzed further. For many lakes, there were records from several different surveys in each period, which sometimes provided contradictory information, perhaps due to low-density roach populations which were undetected in some gillnet surveys. Thus, we excluded all lakes with single observations during periods 1 and 2, and used probabilistic criteria when classifying lakes as acidified or not. Specifically, we classified a lake as acidified if the proportion of surveys that reported roach was ≥75% before 1960 and ≤25% between 1960 and 1990. A lake was classified as nonacidified if more than 90% of the surveys reported roach during both periods. Applying these criteria produced a dataset of 267 lakes, from which 121 were classified as acidified and 146 as nonacidified. Of the total number of lakes that were classified according to the criteria for roach, 85 lakes were also modeled using MAGIC pH reconstruction and those were the lakes used in this study. The 85 lakes covered the natural spatial distribution of roach in Sweden including the southern half and the northern east coast (Fig.1).

### Contemporary water chemistry

**Fig 2:**
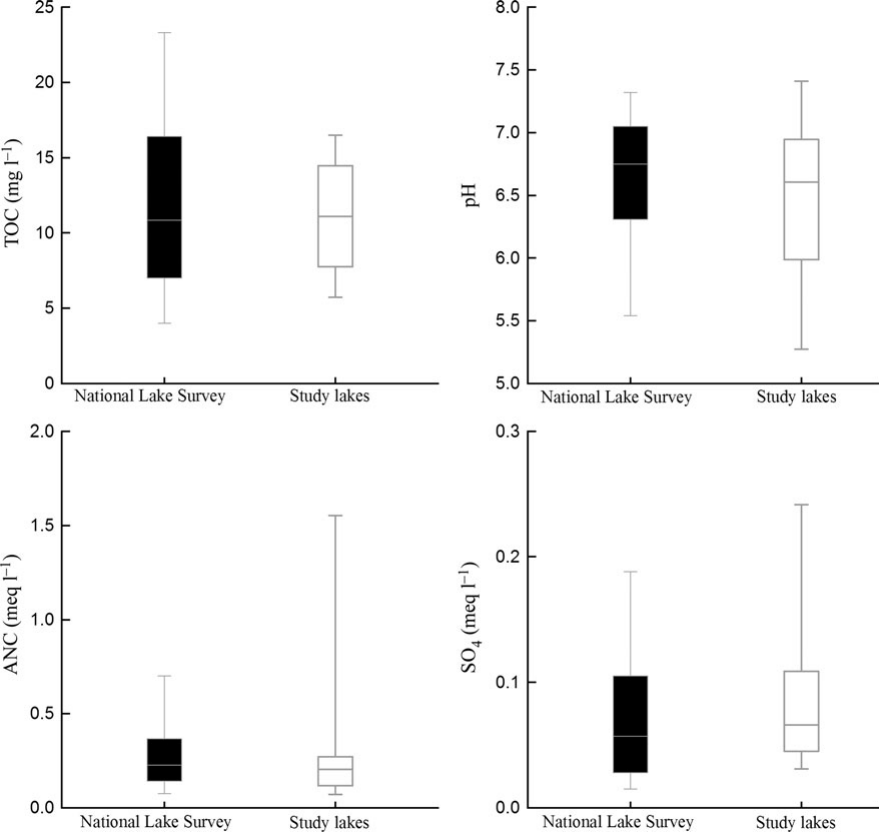
Distribution of chemical parameters in our study lakes (*n* = 85) and in lakes from the national lake survey (*n* = 4600). Measurements were made during 2007–2012. Medians and 5, 25, 75, and 95 percentiles are shown.

All water chemistry data were collected under the Swedish national Lake monitoring program (Fölster *et al*. in prep., http://www.slu.se/vattenmiljo). The 85 lakes in this study covered a wide range of pH, total organic carbon (TOC), and acid neutralizing capacity (ANC). These lakes were compared with 4600 lakes randomly selected during 2007–2012 in Sweden (Fig.2). The 85 lakes included in the study, covered most of the range of a random selection of Swedish lakes (*n* = 4600) in the national lake survey, but with a bias toward acidified lakes. This was reflected in lower pH and ANC and higher SO_4_^2−^ concentrations, although a few lakes with high ANC and pH are included. The pH ranged 4.5 to 8.2, TOC ranged 3 to 32 mg L^−1^_,_ and ANC ranged 0.02 meq L^−1^ to 3.1 meq L^−1^.

### Model of Acidification of Groundwater in Catchments (MAGIC)

The first version of the MAGIC model was developed nearly 30 years ago to quantify and predict effects of acid rain on soils and waters (Cosby *et al.*, 1985). Through the years, the model has been successfully applied to many surface waters in North America, Europe and Asia at numerous monitoring and experimental sites (see Cosby *et al.*, 2001). Typical MAGIC applications involve modeling the temporal evolution of soil and water chemistry from the reference state to the present day using observed or estimated atmospheric deposition and land-use histories at the modeled site. This so-called hindcasting is successfully completed when the reconstructed past leads to simulation of the present-day chemical state of the catchment that matches the observations of soil and water chemistry. At that point, future predictions could be made driven by future scenarios of expected (or hypothetical) air pollution, land use, and climate. The model has been further developed and enhanced in several steps mainly with respect to organic acid buffering (Cosby *et al.*, 1995), Al solubility (Sullivan & Cosby, 1998), nitrogen dynamics (Cosby *et al.*, 2001), and nitrogen and carbon turnover (Oulehle *et al.*, 2012). The MAGIC7 version (Cosby *et al.*, 2001) employed in this work is a lumped-parameter hydrogeochemical model that uses catchment soil characteristics, discharge, precipitation, deposition history, and land-use history as input parameters and driving variables. For model calibration, contemporary water chemistry and soil chemistry were used.

The model simulates monthly or annual concentrations of the major ions in soil solution, streams, or lakes. At the heart of MAGIC is the size of the pool of exchangeable base cations in the soil. As the fluxes to and from this pool change over time, owing to changes in atmospheric deposition, the chemical equilibrium between soil and soil solution shifts to give changes in surface water chemistry (Cosby *et al.*, 1985; Cosby & Wright, 1998). The degree and rate of change in surface water acidity depends on both chemical fluxes and the inherent characteristics of the soils. The mass balance considers mineral weathering, biological uptake and immobilization, atmospheric deposition, decomposition and mineralization of organic matter, and loss to runoff. The equilibrium processes include cation exchange between soil and soil solution, dissolution-precipitation and speciation of inorganic and organically bound aluminum, sulfate adsorption, and speciation of organic and inorganic carbon.

MAGIC has been applied to both individual catchments (Cosby *et al.*, 1985; Kohler *et al.*, 2011; Oulehle *et al.*, 2012) and on regional scales (Evans *et al.*, 1998; Aherne *et al.*, 2003; Moldan *et al.*, 2013). Regional-scale applications typically do not use all the features of MAGIC. For example, MAGIC may be calibrated to only a single point in time or to a reduced subset of parameters. Regional-scale applications of the MAGIC model are used for acidification assessment in Sweden (Moldan *et al.*, 2004) and pH reconstruction for 2985 lakes across Sweden (Moldan *et al.*, 2013).

In the MAGIC simulations presented here, the calibration year with measured water chemistry was set to 2005–2010 (depending on which survey the lake was included in) and we assumed all chemical variables to be at steady state during the reference year of 1860. The reconstructions presented here are based on a regional application of the MAGIC model described by Moldan *et al*. (2013).

### Aluminum in the MAGIC model

In the MAGIC model (Cosby *et al.*, 2001), inorganic Aluminum (Al_i_) dissolution and precipitation are assumed to occur in both the surface water and soil system. This part of the model is driven by two types of reactions: (i) Al_i_ is controlled by equilibrium (K_Al_) with a solid phase of aluminum trihydroxide Al(OH)_3_ and (ii) considering aluminum hydrolysis reactions that involve complexation with sulfate, fluoride, and organic acids. No attempt was made to model complete Al speciation since inorganic aluminum is not regularly analyzed in the Swedish national monitoring program. We therefore excluded fluoride and organic acid complexes in the Al speciation. The median of the solubility constant (K_Al_) employed was 8.1 with a range of 2.4. For the lakes in this study, only six of the 85 lakes required a solubility constant different from 8.1 to correctly predict the observed lake water pH. We used a slope of three in the empirical relationship between pH-pAl (S_Al_) described by (Sullivan & Cosby, 1998). The above procedure allows calculation of the contribution of positive charge of aluminum to the overall water composition, and estimates the presence of inorganic aluminum that is toxic for biota. The equivalent charge contribution of aluminum was calculated as follows: 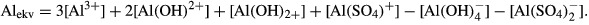
1

Total Al concentrations cannot be estimated using this approach, but this does not affect this study.

### pH in the MAGIC model

The positive charge equivalents in solution as simulated by MAGIC are due to the presence of base cations (Ca^2+^, Mg^2+^, Na^+^, and K^+^), protons, and positively charged aluminum species [Eqn 1]. Negative charge equivalence is due to the presence of acid anions (SO_4_^2−^, Cl^−^, NO_3_^−^, and F^−^) bicarbonate, hydroxide, negatively charged aluminum species [Eqn 1], and organic acids. The dissociation of organic acids is modeled according to Hruška *et al*. (2003).

Bicarbonate was modeled in equilibrium with four times atmospheric pressure (pCO_2_ = 0.156 % by volume). Charge balance was acquired through iteratively adjusting proton concentrations and all proton-related reactions, such as aluminum solubility, bicarbonate, and organic acid equilibrium as well as aluminum speciation. The main charge balance equation was written as follows: 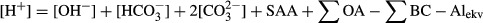
2 where SAA, the strong acid anions, are equal to 2[SO_4_^2-^]+[Cl^-^]+[NO_3_^-^]+[F^-^]; BC is equal to 2[Ca^2+^]+2[Mg^2+^]+[Na^+^]+[K^+^]+[NH_4_^+^]. The organic acids are obtained from the Hruška *et al*. (2003) tri-protic acid calculations ∑ OA=[H_2_A^-^] + 2[HA^2-^] + 3[A^3-^] and ∑ AlF=[AlF_4_^-^] + [AlF_5_^2-^] + 3[AlF_6_^3-^] – 2 [AlF^2+^] – [Al F_2_^+^].

MAGIC is calibrated to observed pH by adjusting the solubility product (K_Al_) of Gibbsite according to the following formula


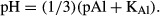
3

where pAl is the negative logarithm (base 10) of the Al^3+^ ion activity. As noted by Cosby *et al*. (2001), there can be considerable variability in the site-specific K_Al_ value. If an erroneous K_Al_ value is used in a MAGIC simulation, estimated pAl will not be representative of in-lake conditions. Despite their large number, the contribution of Al species to the overall charge balance can be neglected when pH is >4.5 (Fig. 5**).** The method of pH calculation in MAGIC is further described in (Cosby *et al.*, 1985, 2001).

## Results

### Comparing chemical and biological measures of acidification and recovery

The comparison between chemical and biological methods for acidification assessment was made for two points in time: 1980 representing the heavy acidification period and 2010 representing the recovery period. To assess the biological effects, two boundaries were considered, ΔpH >0.4 as the classification boundary for good ecological status in Sweden and pH 5.5, which is used as a proxy and threshold value for roach mortality and reproduction failure (Vuorinen *et al.*, 1993; Holmgren & Buffam, 2005).

**Fig 3:**
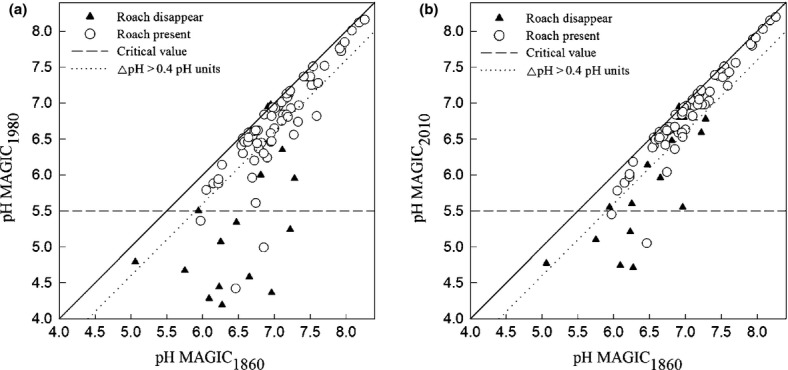
The 85 lakes analyzed with MAGIC hindcasts, and historical observations of roach. On the *Y-*axis, pH reconstructed from MAGIC in 1980 (a) and 2010 (b) and on the *X* axis reconstructed pH 1860. Black triangles (▴) indicate lakes where roach has disappeared and circles (◯) indicate lakes where roach is present. The dotted line shows the criteria for significant acidification (ΔpH > 0.4), the striped horizontal line indicates the critical value of pH 5.5, and the solid black line is the 1/1 line.

MAGIC predicted pH decreased between 1860 and 1980 for all 85 lakes (Fig.3a) and 28 lakes were considered significantly acidified (ΔpH >0.4 units). In 1980, pH was 5.5 or lower for 14 of the acidified lakes and roach were absent in 11. Disagreement between the two methods was found for seven lakes: four where roach was absent and pH >5.5, and three lakes where roach was present and pH <5.5 in 1980 (Fig.3a).

MAGIC predicted chemical recovery for all lakes during 1980–2010 (Fig.3b). At the end of the period, 14 of the 85 lakes were acidified (ΔpH >0.4). Compared to 1980, this was a 50% reduction in the number of acidified lakes. Seven lakes were below pH<5.5 and roach was absent in five (Fig.3b). The chemical recovery was stronger than the recovery of the roach population, 36% of the absent roach populations were found in lakes during the recovery period as a result of active restocking.

A separation of lakes of consistent and inconsistent classification was made. Consistent classification between the two methods occurred in 78 of 85 lakes, and was grouped into two categories: (i) nonacidified in which MAGIC did not predict acidification and roach was present, and (ii) acidified where MAGIC predicted acidification and roach was absent. The remaining seven lakes with inconsistent classification were grouped as: (iii) MAGIC predicted acidification but roach was present and (iv) lakes where MAGIC did not predict acidification but roach was absent.

### Acidification assessment of lakes with consistent classifications

A majority of the lakes (*n* = 64) were classified as nonacidified by both methods and 14 lakes were classified as acidified by both methods. The spatial distribution of these lakes covered large gradients in deposition and climatic conditions (Fig.1). Examples of lakes from each category will be presented in the following sections.

### Lakes predicted as nonacidified by both MAGIC and Roach

**Fig 4:**
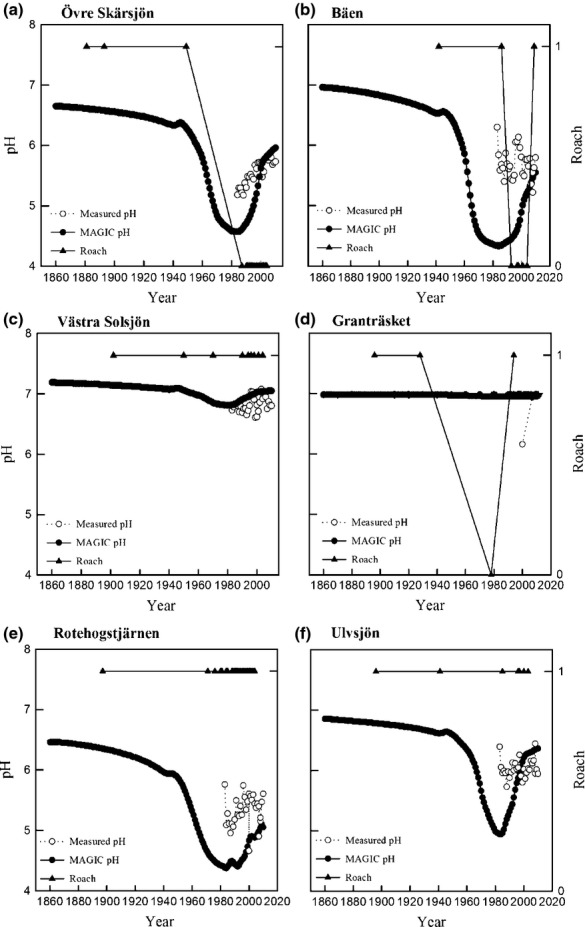
Reconstructed pH from MAGIC and measured pH (left *y*-axis) and presence (1) and absence (0) of roach (right *y*-axis) by year on the *x* axis. (a) Övre Skärsjön. (b) Bäen. (c) Västra Solsjön, (d) Granträsket, (e) Rotehogstjärnen, and (f) Ulvsjön.

In these lakes, acidification was not predicted by MAGIC (ΔpH<0.4) and measured values were above pH 5.5. MAGIC-predicted pH = 7.2 in 1860 and pH = 7.1 in 2010 for Västra Solsjön (Fig.4c). Roach has been present in the lake since the first survey in 1902 (Fig.4c). MAGIC results were similar for Bysjön where pH = 6.8 in 1860 and pH = 6.7 in 2010. Roach has been present since 1896 (data not shown).

### Lakes predicted as acidified by both MAGIC and Roach

In Övre Skärsjön, MAGIC predicted a decrease from pH 6.6 in 1860 to pH 4.7 in 1980, and then an recovery to pH 5.9 in 2010 (Fig.4a). Roach was present in fish surveys until 1950. The historical information states that roach went extinct around 1970 due to acidification from atmospheric deposition and mining activity (Ålind, 1978). In Bäen, MAGIC predicted a decrease from pH 6.9 in 1860, to pH 4.4 in 1980, and pH 5.5 in 2010 (Fig.4b). Roach was absent in the 1994 survey but caught in a survey in 2009 (Fig.4b).

### Acidification assessment in lakes with inconsistent classifications

The two methods gave inconsistent classification in seven lakes. In three lakes, MAGIC predicted acidification but roach was present, and in four lakes MAGIC did not predict acidification but roach was absent.

### Lakes predicted as acidified by MAGIC, but roach was present

MAGIC predicted pH = 6.5 in 1860, pH = 4.4 in 1980, and pH = 5.1 in 2010 for Rotehogstjärnen (Fig.4e). Roach was observed in the lake since 1896. MAGIC-predicted pH=6.9 in 1860, pH=5 in 1980, and pH=6.4 in 2010 for Ulvsjön (Fig.4f). The fit between predicted and observed pH is poor for these lakes (Fig.4e and f).

### Lakes predicted as nonacidified by MAGIC, but roach was absent

MAGIC did not predict acidification for four lakes, but the analysis showed that roach was absent in one or more of the surveys. MAGIC predicted no change in pH for Granträsket, but roach was absent during two surveys in 1977 and 1983 but was identified in 1997 (Fig.4d). MAGIC predicted a pH = 7.1 in 1860 and pH = 7.0 in 2010 for Falträsket. Roach was absent during two surveys in 1980 and 1994 (not shown).

### Measured and modeled inorganic aluminum

**Fig 5:**
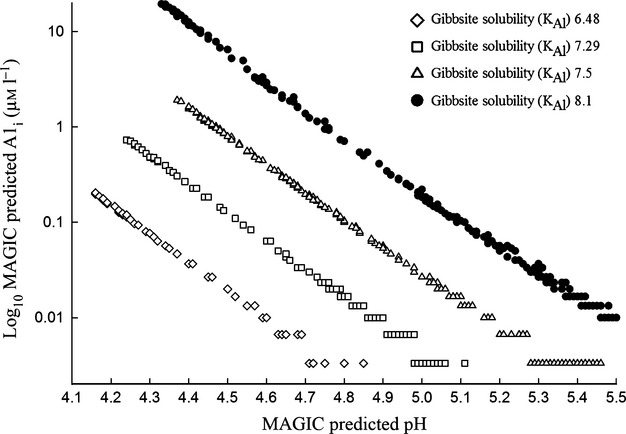
Plot of log_10_ transformed relationship between MAGIC predicted pH and Al_i_ (μmol L^−1^) at a range of gibbsite solubility constants.

**Fig 6:**
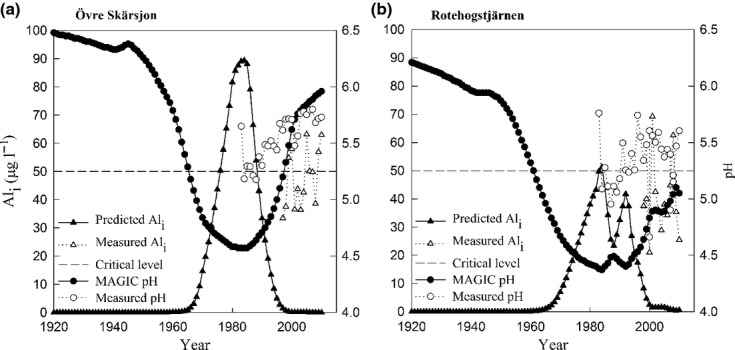
Reconstructed (▴) and measured (Δ) Al_i_ μg L^−1^ on the left axis and reconstructed (●) and measured (◯) pH on the right axis by year. Two lakes with different classifications (a) Övre Skärsjön were both methods agree and (b) Rotehogstjärnen were MAGIC-predicted acidification but roach is present. The dashed horizontal line is the critical level of 50 μg L^−1^ for roach mortality and reproduction failure.

In the regional MAGIC application presented here, no attempt was made to calibrate in-lake Al_i_ concentrations. Long-term (1997–2011), measured Al_i_ values were only available for two lakes in the study set. The highest measured Al_i_ in Övre Skärsjön and Rotehogstjärnen were found in 2001, with 51 and 69 μg L^−1^, respectively (Fig. 6). Övre Skärsjön exceeded the critical limit of 50 μg L^−1^ once since 1997, whereas Rotehogstjärnen exceeded the limit multiple times. To further elaborate the specific effects of Al speciation on biota, the MAGIC model needs to be calibrated toward a large dataset. In our application of the MAGIC model, given the assumptions made for Al_i_, ten of the 85 lakes predicted Al_i_ > 1 μg L^−1^. Three lakes predicted Al_i_ > 50 μg L^−1^. MAGIC predicted Al_i_ to 90 μg L^−1^ between 1980 and 1990 for Övre Skärsjön (Fig. 6a). The modeled Al_i_ for Rotehogstjärnen does not exceed 50 μg L^−1^ during 1980–1990 (Fig. 6b). The critical Al_i_ level for roach (50 μg L^−1^) does not occur when pH is above 4.8 (Fig.5).

### Uncertainty in MAGIC and roach datasets

The uncertainty for the two datasets was evaluated with contemporary measured values. MAGIC was calibrated to the year 2010 for most lakes. A comparison between measured and predicted pH for the calibration year resulted in a mean absolute error of 0.07 pH units. Comparing the long-term average for all lakes (1983–2010) resulted in a mean absolute error of 0.4 pH units, and the largest mean absolute error for a single year (1983) was 0.8 pH units. Some individual lakes had poor agreement between measured and predicted pH during 1984–2010 (e.g., Fig.4b, e, f). Nonacidified lakes by MAGIC (*n* = 71) had a mean absolute error of 0.3 pH units. Acidified lakes by MAGIC (*n* = 14) had a mean absolute error of 0.6 pH units.

The TOC concentration was evaluated for the four classes. No statistically significant difference was identified. The median TOC was 9 ± 2 mg L^−1^ (±SD) for all lakes. In the acidified lakes by both methods, the median TOC was 8 ± 3 mg L^−1^, and lakes classified as nonacidified by both methods, TOC was 9 ± 4 mg L^−1^. In lakes where roach was absent and not acidified by MAGIC, TOC was 8 ± 5 mg L^−1^. In lakes where MAGIC predicted acidification but roach were present, TOC was slightly higher, 11 ± 3 mg L^−1^.

The roach dataset was analyzed for data availability and reliability. Many historical observations were presented in this dataset through surveys and gillnet fishing. In the analysis, data from two periods were prioritized, ‘beginning of acidification period’ <1960 and ‘heavy acidification period 1960–1990’. Data after 1990 were not as comprehensive as for the other two periods, but still important for addressing the recovery (Table1). It was shown that false absences and notes on species introduction were limitations to this approach.

**Table 1:** Number of surveys and observations of roach for each of the three periods for roach: beginning of acidification period, heavy acidification period, and recovery period

Sampling period	No. of surveys	Lakes with Single observation	Lakes with Two observations	Lakes with Three or more observations
Beginning of acidification period <1960	237	33	23	29
Heavy acidification period 1960–1990	219	32	22	31
Recovery period >1990	170	55	11	29

## Discussion

The straightforward comparison of hydrogeochemical modeling and presence/absence of fish provided a greater understanding of a century of anthropogenic pressure and provided an alternative reference condition in boreal lakes. Our results show that the combination of hindcast models and historical fish species composition gave valuable information on long-term effects of anthropogenic impact. The unique dataset on presence and absence of roach presented in this study improved the understanding of biological effects from acidification and will be of great value for large parts of Europe, North America, and Asia.

### Lakes with consistent classification

Previous attempts to validate biological and chemical predictions of reference conditions have been made with paleolimnology in Sweden (Erlandsson *et al.*, 2008a), Europe (Battarbee *et al.*, 2005), and North America (Wright *et al.*, 1986). One of the main limitations of diatom reconstructions is the amount of time needed to reconstruct one single lake to estimate the anthropogenic pressure, which in a country like Sweden, with 100 000 lakes could be very demanding. We have shown that long-term records of acid-sensitive fish species, after presence and absence analysis were highly coherent with hindcast reconstructions of surface water pH. The high coherency between the two methods validates the use of historical fish species composition as a tool to estimate anthropogenic pressure. We used acidification as an example, and we are convinced that this approach can be used with other species to address other environmental concerns in other regions than Sweden, such as; climate change (Hein *et al.*, 2012), effects of land alterations (Nilsson *et al.*, 2005; Labay *et al.*, 2011), and other airborne pollutants.

The analysis showed that 64 lakes were not classified as acidified using either MAGIC or roach observations (Fig.3a and b, 4c). These lakes included both acid-sensitive lakes in northern low deposition areas, and well buffered lakes in southern Sweden. The large variety of lakes demonstrates that roach is a stable indicator for anthropogenic acidification across the boreal region (Fig.1). The 14 anthropogenically acidified lakes with consistent classification (Fig.3a and b, 4a and b) were located in highly affected areas in south and southwest Sweden, where roach was extirpated due to high rates of sulfur deposition. We have shown with a large number of lakes that roach is a good indicator of the historical changes and can be used in regional assessment of acidification.

### Lakes with inconsistent classification

#### Lakes Classified as acidified by roach, but not by MAGIC

Four lakes were not acidified by MAGIC, but roach was absent in one or more survey while measured and predicted pH was above the critical value (Fig.4d). The probable hypothesis for this misclassification was false absences of roach and/or low-density roach populations. False absences might occur with historical and present questionnaires, and single surveys increase the possibility for low-density roach communities to go undetected. For instance, generalized grouping of cyprinids into ‘whitefish’, which usually includes roach, has occurred in this dataset. If roach classed as ‘whitefish’, a false absence would have occurred and lakes would have been incorrectly classified as acidified. This uncertainty was managed by discussions with fishery conservation organizations, local landowners, and anglers. Consultation with local experts gave us knowledge that roach had been present throughout time, and thus these lakes had false absences of roach. Local knowledge has proven important to identify reference conditions and understand historical changes in water quality (Valinia *et al.*, 2012). With local experts, we could reclassify these lakes as nonacidified. However, keeping these lakes in this category highlights the uncertainties and false absences that might occur when using long-term biological databases.

#### Lakes Classified as acidified by MAGIC, roach is present

MAGIC predicted acidification but roach was present in three lakes, a probable hypothesis was underestimation of pH in the hindcast modeling compared to contemporary measured values. The 3 lakes were included in the Swedish monitoring program and chemical parameters were available since 1983 (Fig.4e and f). The fit between predicted pH and measured mean annual pH was poor, mean absolute error = 0.8 pH units for Ulvsjön (Fig.4f) and Rattsjön (not shown). These two lakes had no visible trend in measured pH and were clearly above the critical value of 5.5, while MAGIC predicted a substantial recovery since 1980 (Fig.4e). An even larger error was identified for Rotehogstjärnen with mean absolute error = 1.1 pH (Fig.4e). However, the measured trend was similar to the trend predicted by MAGIC and measured pH was around the critical value of 5.5 (Fig.4e). The reconstructed absolute values for these three lakes could be disregarded due to the substantial discrepancy in measured and predicted pH. One possible explanation for this discrepancy can be the relatively high TOC concentrations. While it is not possible to say anything statistically meaningful with such a small sample size (*n* = 3); TOC in these lakes was higher (11 ± 3 mg L^−1^) than the overall average (9 ± 2 mg L^−1^). TOC has been increasing in surface waters across Europe and North America (Monteith *et al.*, 2007) and changes have been identified on millennial and centennial timescales (Rosen, 2005; Cunningham *et al.*, 2011; Rosén *et al*., 2011; Valinia *et al.*, 2012). Suggested mechanisms for the increasing trend are: recovery from acidification (Evans *et al.*, 2005), changes in temperature (Freeman *et al.*, 2001), precipitation (Ledesma *et al.*, 2012) and hydrological influences in combination with acidification recovery (Erlandsson *et al.*, 2008b). In certain pH intervals, a small change in TOC can influence pH substantially (Köhler *et al*., 2000), and discrepancies between measured and predicted pH in the MAGIC model can partly be explained by changes in TOC (Erlandsson *et al.*, 2008a, 2011). The MAGIC model was calibrated to one measurement, i.e. the calibration year. The calibration year TOC was 16 ± 1 mg L^−1^ whereas the long-term (1984–2010) average was 11 ± 3 mg L^−1^. Higher TOC leads to greater amounts of organic acidity, and if the calibration year were unrepresentative, the hindcast modeling would not reproduce the trend of measured pH (Fig.4e and f). These results show the importance of organic acids when estimating reference conditions, hence, acidification assessment in boreal lakes.

Adding to the complexity was the potential for biological lag time as a response to acidification. Biological lag time was evident for Rotehogstjärnen and Bäen (Fig.4b and e). Both lakes had an increase in mean size and an older population age structure for roach since late 1980. These factors were identified as a response to acidification-induced reproductive failure (Tammi *et al.*, 2004; Holmgren & Buffam, 2005; Dahlberg, 2006). Adult roach managed to survive in Rotehogstjärnen, while it was extirpated in Bäen late 1990. These two lakes were good examples of biological lag times and the response to anthropogenically induced change can vary quite substantially.

### Inorganic aluminum as a cause of discrepancy between the two methods

Only two of the 85 lakes had measured values of Al_i,_ and drawing general conclusions about the effect of Al_i_ in acidified lakes was not possible. Because the MAGIC simulations presented here were not calibrated to measured values, the uncertainties of Al_i_ are large, even though it was possible to obtain acceptable simulations of pH in 79 of 85 lakes using the same Gibbsite solubility constant [Fig.5 and Eqn 3]. The well established relationship between pH and Al_i_ in slightly acidic to near neutral surface waters (Sjöstedt *et al*., 2010) motivates the assumption that pH can be used as the proxy for the toxic effects on biota when Al_i_ measurements are missing. Övre Skärsjön was acidified with both methods and measured Al_i_ was around the critical limit of 50 μg L^−1^ or below (Fig.6a). Rotehogstjärnen was acidified (ΔpH>0.4), Al_i_ levels were higher than 50 μg L^−1^ on multiple occasions but roach was present throughout time. In this case, Al_i_ may not be a determining factor for roach extinction. The higher organic carbon can also be a factor for the discrepancies because of its tendency to bind with organic matter, Al is generally less toxic than at equivalent concentrations in clear water lakes (Driscoll & Schecher, 1990).

### Biological and chemical recovery from acidification

All lakes had shown some degree of chemical recovery in the year 2010 compared to 1980 (Fig.4a and b) and multiple studies have identified the same trend in Europe and North America. (Stoddard *et al.*, 1999; Skjelkvåle *et al*., 2005; Larssen *et al.*, 2010). A classification change based on the ΔpH method occurred in 50% of the lakes between 1980 and 2010 (Fig.3a and b), which by itself demonstrates the efficacy of measures to reduce acidifying emissions. On the other hand, evidence of biological recovery is not as clear as for chemical recovery. After 1990, only five lakes from which roach was earlier extirpated had reproducing populations. The first roach extirpation was documented in 1940 for an acidified lake and the latest in 1994 (Fig.4b) while the roach population (Fig.4e) was present in Rotehogstjärnen throughout the acidification period. Chemical recovery to an acceptable status means the potential exists for biological recovery to occur. However, for example, episodic acidification, not covered by extensive monitoring programs of water chemistry, may prevent or delay a biological recovery (Kowalik *et al.*, 2007). Other nonchemical factors including water temperature (Johnson & Angeler, 2010) or species interactions (Yan *et al.*, 2003) may also be important. We can estimate the variability in responses of roach to acidification in boreal lakes. Natural re-establishment of roach is very slow, and to our knowledge, none of these lakes has a natural pathway for recolonization. Discussions with anglers and other experts suggest that successful roach recolonization is most likely in lakes where active restocking has occurred. Using hindcast modeling and historical data, we can obtain a greater understanding of regional recovery from anthropogenic acidification and biological lag times in boreal lakes and elsewhere.

### Conceptual Model

**Fig 7:**
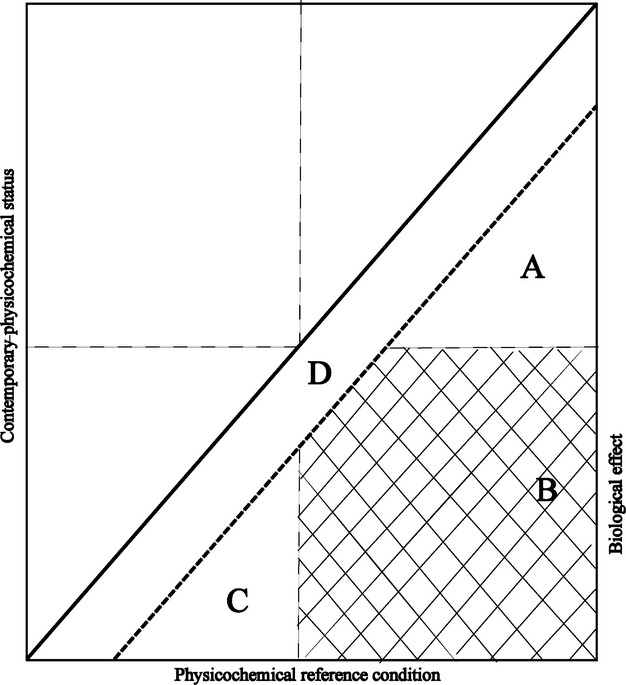
A conceptual model showing how information about physico-chemical reference conditions and historical species data can be used to guide management actions. The thick diagonal 1/1 line represents the correspondence between reference and assessment period. The thick dashed line represents the accepted deviation based on physico-chemical criteria. The thin dashed vertical and horizontal line represents physico-chemical threshold for presence of the target organism. Area D is within the accepted deviation from a reference state, whereas in area A and C no biological effect will occur and in area B a significant effect on target organism occurs.

The novel results suggest a conceptual model for making priorities of measures when there is an effect on a valuable indicator species. This model was derived from the assessment of lake acidification, but we propose that a conceptual model based on Fig.3a and b can be used for understanding the effects of human stressors on the natural environment more generally (Fig.7). The region where significant anthropogenic effects are suggested as a result of deviations from reference conditions based on physico-chemical criteria can be divided into four parts: (i) there is no effect on the chosen species as the change in physico-chemical environment was too small to have any ecologically meaningful effect on the biological indicator; (ii) substantial chemical change where biological conditions shifted from favorable to unfavorable; (iii) no effect on the chosen species because conditions during the reference state were unfavorable for the species; (iv) an accepted deviation from reference conditions. This conceptual model can be applied more generally using a positive deviation from reference conditions (iv) and a higher threshold based on physico-chemical criteria. This includes parameters (e.g., temperature, nutrients, and metals) where higher values are usually considered negative for the biological target organisms.

### Policy implications

Undisturbed state or reference conditions were designed to protect the ecosystem regardless of the socioeconomic value. In the implementation of water legislation, like the WFD and Clean Water Act, there is a need to prioritize the remediation and restoration efforts to sites where the costs can be justified toward stakeholders and by benefits. The conceptual model presented here (Fig.7) can be a valuable tool if the threshold value is set according to a species that is regarded as valuable to society, for example a fish species. While the criteria of a change in pH of 0.4 units used in the Swedish EQC was designed to protect all organisms, the conceptual model presented here helps policy makers to focus the measures to sites having a potential to hold species regard valuable to the public (region B). There is less value in restoring reference condition levels in surface waters where valuable species were never present (region C) or where the change in water chemistry is not sufficient to threaten the species (region A).

A change in the recommendations about how we manage water bodies concerning restorations, reference conditions, and ecological integrity should be considered (Fig.7). By clearly focusing on impacted water bodies where remediation to a state approaching reference conditions is both possible and desirable for stakeholders, we can effectively use all available knowledge, including expert and local, to successfully and sustainably manage our surface waters.
